# Private Equity Acquisitions of Home Health Agencies

**DOI:** 10.1001/jamahealthforum.2025.4922

**Published:** 2025-11-14

**Authors:** David T. Zhu, Aakash Reddy, Geronimo Bejarano, Robert Tyler Braun

**Affiliations:** 1 Medical Scientist Training Program, School of Medicine, Virginia Commonwealth University, Richmond; 2 Weill Cornell Medical College, New York, New York; 3 Department of Health Services, Policy, and Practice, Brown University, Providence, Rhode Island; 4 Department of Population Health Sciences, Weill Cornell Medicine, New York, New York; 5 Cornell Health Policy Center, Weill Cornell Medicine, New York, New York

## Abstract

This cross-sectional study examines the pattern of home health agency acquisitions by private equity in the US by year, fund size of the acquiring firm, and location.

## Introduction

Postacute and long-term care delivered in US home health agencies (HHAs) has expanded over the past decade, driven by an aging population and efforts to shift care from hospitals to lower-cost settings.^1^ In parallel, HHAs have become increasingly attractive to institutional investors. Private equity (PE) investment in health care has increased across multiple health care sectors, reshaping care delivery and market structure.^2^ Rising demand and the industry’s fragmented landscape have drawn PE attention to HHAs, where roll-up strategies (acquiring and consolidating smaller agencies into larger firms) can increase valuations through multiple arbitrage.^1,3^ This study aimed to describe patterns in PE acquisitions of HHAs.

## Methods

Using established methodologies,^3^ we identified PE acquisitions of HHAs from 2006 to 2024 in the Irving Levin health care market database, verified by industry reports, press releases, and HHA websites (eMethods 1 in Supplement 1). The Weill Cornell Medicine Institutional Review Board approved this cross-sectional study and waived informed consent because deidentified data were used. We followed the STROBE reporting guideline.

Provider of Services files were used to obtain each HHA’s Centers for Medicare & Medicaid Services (CMS) Certification Number, location, and other HHAs in the same transaction. We calculated the percentage of acquisitions by year, PE fund size, top-10 PE acquirers and acquired HHAs, Census region, and state. PE fund size was obtained from firms’ websites and categorized as lower-middle market ($20-$100 million), middle market (>$100-$500 million), upper-middle market (>$500 million-$1 billion), or megafund (>$1 billion). Prior evidence suggests fund size changes PE acquisition strategies and target selection^4^; thus, we examined whether HHA acquisitions were concentrated in certain funds or broadly distributed (eMethods 2 in Supplement 1). Data analysis was performed with R, version 4.3.3 (R Core Team).

## Results

From 2006 to 2024, we identified 749 unique HHAs, of which 55 (7.3%) were involved in secondary PE buyouts (Table). Most HHAs were acquired by middle-market (520 [69.4%]) or megafund (143 [19.1%]) PE firms. Acquisitions tended to occur in batches, particularly in 2017 (156 [20.8%]), 2018 (162 [21.6%]), and 2021 (283 [37.8%]) (Figure, A). Megafunds accounted for most acquisitions in 2017, while middle-market firms led in 2018 and 2021.

**Table.** Private Equity Acquisitions of Home Health Agencies

Characteristic	Total No. (%) (N = 749)^a^
Reacquisitions	
Total	55 (7.3)
1	49 (6.5)
2	3 (0.4)
Target home health agency^b^	
Caring Brands International/Interim HealthCare	258 (34.4)
Elara Caring	96 (12.8)
Aveanna Healthcare	84 (11.2)
Just Home Healthcare Services	34 (4.5)
BrightSpring Health Services	33 (4.4)
Concierge Home Care	25 (3.3)
Team Select Home Care	22 (2.9)
Healthy Living at Home	18 (2.4)
Choice Health at Home	14 (1.9)
AccentCare Home Health	13 (1.7)
Other^c^	152 (20.3)
Private equity fund size, $^b^	
Lower-middle market: 20 to 100 million	85 (11.3)
Middle market: >100 to 500 million	520 (69.4)
Upper-middle market: >500 million to 1 billion	1 (0.1)
Megafund: >1 billion	143 (19.1)
Private equity firm^b^	
Wellspring Capital Management	263 (35.1)
Blue Wolf Capital	96 (12.8)
Bain Capital	89 (11.9)
H.I.G. Capital	34 (4.5)
Kohlberg Kravis Roberts & Co	33 (4.4)
Waud Capital Partners	25 (3.3)
Tenex Capital Management	22 (2.9)
Capricorn Healthcare	18 (2.4)
Trive Capital	14 (1.9)
Advent International	13 (1.7)
Other^d^	142 (19.0)
US Census region^b^	
South	355 (47.4)
Midwest	172 (23.0)
West	118 (15.8)
Northeast	104 (13.9)
State^b^	
Florida	115 (15.4)
Texas	85 (11.3)
California	67 (8.9)
Pennsylvania	43 (5.7)
New Jersey	36 (4.8)
Indiana	32 (4.3)
Oklahoma	29 (3.9)
Kentucky	28 (3.7)
Colorado	26 (3.5)
Ohio	26 (3.5)
Other^e^	262 (35.0)

^a^ Percentages may not sum to exactly 100% due to rounding.

^b^ Variable current at the time of the index acquisition.

^c^ Other included Abode Healthcare, Accurate Home Care, Advantage Nursing Services, Aging With Comfort, All Metro Health Care Services, Amazing Care Home Health Services, AmeriBest Home Care, American HomePatient, Angels of Care, Arosa, BrightStar Care, Care Advantage, CarePeople Home Health, Central Home Health Services of Texas, Charter Healthcare Group, ComForCare Health Care Holdings, Comfort Keepers, Coordinated Home Care, Encompass Health, EPIC Homehealth Services, Executive Home Care Franchise, Family Tree Private Care, For Papa’s Sake Home Care, Great Lakes Caring Home Health and Hospice, HarmonyCares, Help at Home, Helping Hands Home Care, Home Helpers Home Care, Hometown Hospice and Homecare, HouseWorks, InnovAge, Integracare Home Health, Interim HealthCare, Jordan Health Services, LifeCare Home Health Family, Matrix Medical Network, Maverick Healthcare, MGA Homecare, Mission Healthcare, National Home Health Care, Pediatric Home Service, Pediatric Services of America, Providence Care, Senior Home Care Inc, Sona's Homecare, Southeastern Home Health Care, Superior Health Holdings, Synergy HomeCare, The Care Team, THEMA Health Services, Valeo Home Health and Hospice, Visiting Nurse Association, and Willcare.

^d^ Other included Actinium Healthcare Holdings; Alpine Investors; Angelo, Gordon & Company; Beecken Petty O'Keefe & Company; BelHealth Investment Partners; Bow River Capital; Boyne Capital Partners; Firmament Group; Flexpoint Ford; Frazier Healthcare Partners; Generation Growth Capital; Geneva Glen Capital; Grant Avenue Capital; Great Point Partners; Halifax Group; Havencrest Capital Management; Highland Capital Management; InTandem Capital Partners; InvestSouth; J.H. Whitney Capital Partners; JPB Partners; Linsalata Capital Partners; Nautic Partners; NexPhase Capital; Oaktree Capital Management; Palladium Equity Partners; Pharos Capital Group; Portfolio Logic Management; Revelstoke Capital Partners; Riverside Company; Rubicon Founders; Silver Oak Services Partners; Summer Street Capital Partners; Syndicate Capital; Tailwind Capital; Thoma Cressey Bravo; Transition Capital Partners; Varsity Healthcare Partners; Vistria Group; Webster Equity Partners; Welsh, Carson, Anderson & Stowe; and Zenyth Partners.

^e^ All states except Mississippi, Vermont, and Alaska. Hawaii had 6 private equity acquisitions of home health agencies, but it is not shown in the choropleth map (Figure, B).

**Figure.**
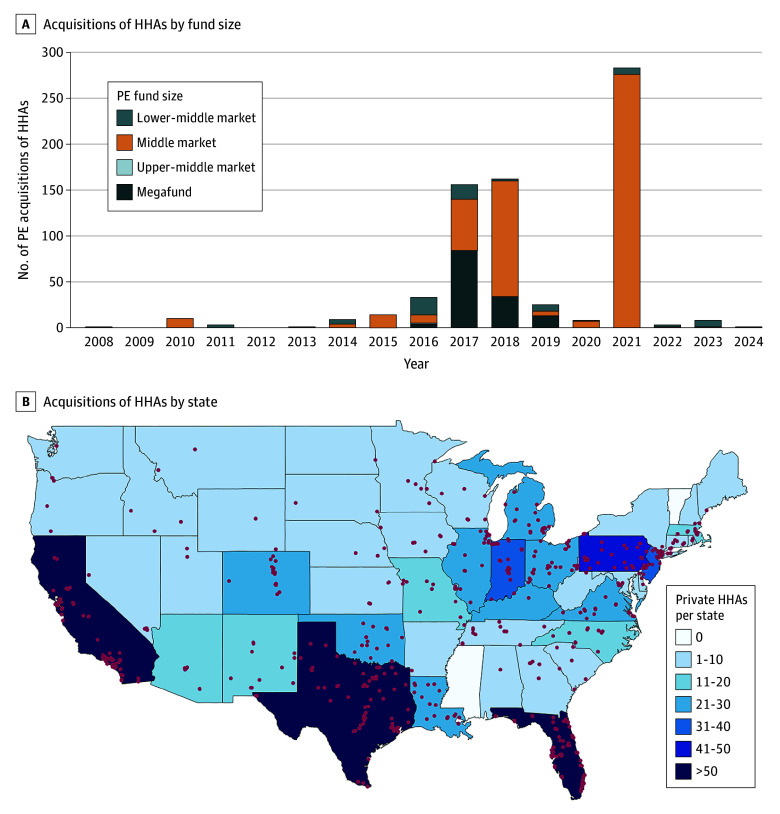
Private Equity (PE) Index Acquisitions of Home Health Agencies (HHAs) by PE Fund Size and State From 2006 to 2024 A, Lower-middle market fund size: $20 to $100 million, middle market fund size: more than $100 to $500 million, upper-middle market fund size: more than $500 million to $1 billion, and megafund size: more than $1 billion. B, Overlying dots indicate the county where each HHA involved in a PE acquisition was located.

We identified 63 distinct HHA chains and 52 PE firms (Table). Most acquisitions involved just 3 target HHAs: Caring Brands International/Interim HealthCare (258 [34.4%]), acquired by Wellspring Capital Management in 2021; Elara Caring (96 [12.8%]), acquired by Blue Wolf Capital in 2018; and Aveanna Healthcare (84 [11.2%]), acquired by Bain Capital in 2017.

Regionally, most acquisitions occurred in the South (355 [47.4%]), followed by the Midwest (172 [23.0%]), West (118 [15.8%]), and Northeast (104 [13.9%]) (Figure, B). Florida accounted for the largest share (115 [15.4%]), followed by Texas (85 [11.3%]) and California (67 [8.9%]).

## Discussion

This study extends prior research on PE acquisitions in hospitals, hospices, and nursing homes by examining the HHA sector.^1,2^ PE acquisitions of HHAs accelerated after 2017, led by middle-market and megafund firms, reflecting sustained interest across a broad range of PE investors. Regional concentration in the South, particularly Florida and Texas, parallels PE activity in other health care sectors and aligns with regions experiencing rising demand for HHAs due to aging populations.^2^

Policy changes may have contributed to these PE acquisition patterns, including the 2016 statewide extensions of CMS-imposed moratoria in Florida and Texas (which restricted new HHA market entrants) and the 2017 repeal of the proposed Home Health Groupings Model (which would have substantially reduced future Medicare payments to HHAs).^5,6^ Further research is needed to examine how PE fund size and evolving policy environments affect investment strategies in HHAs.

Study limitations include possible omission of smaller or unreported transactions and exits or divestitures, which may have shifted the composition of PE-owned HHAs over time. Future research should expand on this descriptive work to evaluate the association of PE ownership with quality of care, patient outcomes, market competition, and spending in HHAs.

## Notes

Supplement 1.
eMethods 1.

eMethods 2.

eReferences.

Supplement 2.
Data Sharing Statement
