# Supplementary material for: Private Equity Acquisitions of Home Health Agencies
Source: JAMA Health Forum. 2025 Nov 14;6(11):e254922. doi: 10.1001/jamahealthforum.2025.4922 (PMC12619096; doi:10.1001/jamahealthforum.2025.4922)
Supplement: Supplement 1. — eMethods 1. eMethods 2. eReferences. [file jamahealthforum-e254922-s001.pdf]

Supplemental Online Content

Zhu DT, Reddy A, Bejarano G, Braun RT. Private equity acquisitions of home health agencies. *JAMA Health Forum*. 6(11):e254922. doi:10.1001/jamahealthforum.2025.4922

eMethods 1

eMethods 2

eReferences

This supplemental material has been provided by the authors to give readers additional information about their work.

© 2025 Zhu DT et al. *JAMA Health Forum*

eMethods 1

We conducted a cross-sectional analysis of private equity (PE) acquisitions of Medicare-certified home health agencies (HHAs) in the United States between 2006 and 2024. Acquisitions were identified using the Irving Levin Healthcare Market Database, a leading source for transaction-level data on healthcare consolidation.[1] This database compiles information from financial disclosures, proprietary deal reports, and press releases, and is well suited for studying PE activity in fragmented sectors such as home health, where many firms are privately held and lack uniform public reporting. PE involvement was identified using LevinPro HC's transaction classification, which flags acquisitions made by a PE firm or a PE-backed portfolio company. PE firms are investment entities that pool capital from external investors and uses the funds to acquire ownership stakes in companies, typically using a combination of investor-contributed equity and debt financing secured against the target's assets or projected earnings.

We constructed a facility-level analytic dataset by linking acquired HHAs to Centers for Medicare & Medicaid Services (CMS) Provider of Services (POS) files using a process adapted from prior work on hospice acquisitions by PE firms and publicly traded corporations.[1] Although hospice agencies and HHAs differ in certain regulatory and operational respects (e.g. scope of services, patient populations), both are Medicare-certified post-acute care providers with unique CMS Certification Numbers (CCNs) and comparable facility-level reporting requirements, allowing similar investigative strategies to be applied.

Acquisitions from the Irving Levin Healthcare Market Database were linked to the POS by searching agency names and confirming that the CCN and address matched those in industry sources or on the agency's website. Acquisitions involving agencies that only provided durable medical equipment, infusion therapy, or hospice/palliative care were excluded. If no match was found in the most recent POS, historic POS files from the acquisition year were reviewed to account for potential name changes, and online searches were conducted to identify alternate names. Addresses were verified by comparing POS-listed locations with those in industry reports or on agency websites. This process yielded 749 verified CCNs from 2006 to 2024, and all matches were manually validated through official website checks and online location searches to confirm they corresponded to active HHAs under PE ownership.

© 2025 Zhu DT et al. *JAMA Health Forum*

eMethods 2

PE fund size was defined based on self-reported assets under management or fund sizes obtained from PE firm websites and classified into four tiers using established investment banking benchmarks: lower-middle market ($20-$100 million), middle market ($100-$500 million), upper-middle market ($500 million-$1 billion), and megafund (over $1 billion).

We included PE fund size in our analysis based on literature showing that fund scale influences acquisition strategy, deal structure, and post-acquisition growth models.[2,3] Larger funds, with greater access to capital, tend to acquire larger platform companies and expand them through multiple add-on acquisitions, leveraging economies of scale and consolidation to increase valuations, including through multiple arbitrage.[2,3] Smaller and middle-market funds frequently execute numerous smaller add-on acquisitions, which can produce regional clustering and localized consolidation, even without a single dominant platform.[4] In fragmented healthcare markets such as HHAs, where many agencies are small in scale, variation in fund size can influence both the pace and extent of market concentration as well as the potential for regulatory scrutiny.[5] Ongoing policy attention to serial "roll-up" strategies, particularly those executed through transactions below federal reporting thresholds,[6,7] highlights the relevance of distinguishing investors by fund size when characterizing acquisition trends. For these reasons, we examined whether HHA acquisitions were concentrated among certain funds or broadly distributed. Future work should examine heterogeneity across transactions and broader market forces (e.g., interest rates), as variation in deal-level and macroeconomic conditions may influence acquisition strategies and market concentration.

eReferences

1. Braun RT, Stevenson DG, Unruh MA. Acquisitions of Hospice Agencies by Private Equity Firms and Publicly Traded Corporations. *JAMA Intern Med*. 2021;181(8):1113-1114. doi: 10.1001/jamainternmed.2020.6262.
2. Hammer B, Knauer A, Pflücke M, Schwetzler B. Inorganic growth strategies and the evolution of the private equity business model. *J Corp Finance*. 2017;45:31-63. doi: 10.1016/j.jcorpfin.2017.04.006.
3. Hammer B, Marcotty-Dehm N, Schweizer D, Schwetzler B. Pricing and value creation in private equity-backed buy-and-build strategies. *J Corp Finance*. 2022;77:102285. doi: 10.1016/j.jcorpfin.2022.102285.
4. American Investment Council. Private Equity and Main Street: An Outlook on the Middle Market. Published on September 18, 2024. Accessed on August 14, 2025. https://www.investmentcouncil.org/wp-content/uploads/2024/09/Report-Private-Equity-Main-Street_An-Outlook-on-the-Middle-Market-September-2024.pdf
5. Zhang Z, Li K, Wang S, Fashaw-Walters S, Hou Y. Change of Ownership and Quality of Home Health Agency Care. *JAMA Health Forum*. 2024;5(11):e243767. doi:10.1001/jamahealthforum.2024.3767
6. Cai C, Song Z. A Policy Framework for the Growing Influence of Private Equity in Health Care Delivery. *JAMA*. 2023;329(18):1545-1546. doi: 10.1001/jama.2023.2801.
7. Federal Trade Commission. FTC and DOJ Seek Info on Serial Acquisitions, Roll-Up Strategies Across U.S. Economy. Published on May 23, 2024. Accessed on August 14, 2025. https://www.ftc.gov/news-events/news/press-releases/2024/05/ftc-doj-seek-info-serial-acquisitions-roll-strategies-across-us-economy
