# Supplementary material for: Private Equity Acquisitions of Home Health Agencies
Source: JAMA Health Forum. 2025 Nov 14;6(11):e254922. doi: 10.1001/jamahealthforum.2025.4922 (PMC12619096; doi:10.1001/jamahealthforum.2025.4922)
Supplement: Supplement 2. — Data Sharing Statement [file jamahealthforum-e254922-s002.pdf]

Data Sharing Statement

Zhu. Private Equity Acquisitions of Home Health Agencies. *JAMA Health Forum*. Published November 14, 2025. doi:10.1001/jamahealthforum.2025.4922

Data
Data available: No
